# Coherent control of double deflected anomalous modes in ultrathin trapezoid-shaped slit metasurface

**DOI:** 10.1038/srep37476

**Published:** 2016-11-22

**Authors:** Z. Zhu, H. Liu, D. Wang, Y. X. Li, C. Y. Guan, H. Zhang, J. H. Shi

**Affiliations:** 1 Key Laboratory of In-Fiber Integrated Optics of Ministry of Education, College of Science, Harbin Engineering University, Harbin 150001, China; 2 SZU-NUS Collaborative Innovation Centre for Optoelectronic Science & Technology, and Key Laboratory of Optoelectronic Devices and Systems of Ministry of Education and Guangdong Province, Shenzhen University, Shenzhen, China

## Abstract

Coherent light-matter interaction in ultrathin metamaterials has been demonstrated to dynamically modulate intensity, polarization and propagation direction of light. The gradient metasurface with a transverse phase variation usually exhibits an anomalous refracted beam of light dictated by so-called generalized Snell’s law. However, less attention has been paid to coherent control of the metasurface with multiple anomalous refracted beams. Here we propose an ultrathin gradient metasurface with single trapezoid-shaped slot antenna as its building block that allows one normal and two deflected transmitted beams. It is numerically demonstrated that such metasurface with multiple scattering modes can be coherently controlled to modulate output intensities by changing the relative phase difference between two counterpropagating coherent beams. Each mode can be coherently switched on/off and two deflected anomalous beams can be synchronously dictated by the phase difference. The coherent control effect in the trapezoid-shaped slit metasurface will offer a promising opportunity for multichannel signals modulation, multichannel sensing and wave front shaping.

Metasurface as 2D metamaterial has received increasing interest and is a promising candidate for manipulating amplitude, phase and polarization of electromagnetic wave with a transverse phase variation along the interface^1^,^2^,^3^. Metasurfaces hold attractive advantages such as ultrathin layer, compact size, low cost and easy-of-fabrication. When light is incident on a metasurface with a gradient phase change along the surface, anomalous reflection and refraction are allowed and dominated by a so-called generalized form of Snell’s law^1^. Subsequently, tremendous efforts have been devoted to the exploration of gradient metasurfaces, leading to the demonstration of vortex plate^1^, wave front shaping^4^, photonic spin Hall effect^5^, a propagating-to-surface-wave converter^6^, flat and achromatic lenses^7^,^8^,^9^ and high efficient optical holograms^10^. As one of the most exciting applications, metamaterial-based absorbers have acquired considerable attention and realized weak radiation and nearly total absorption^11^. Multiband, polarization-insensitive, angle-independent and tunable absorbers have continuously been demonstrated in microwave, THz and optical range^11^,^12^,^13^,^14^,^15^,^16^,^17^,^18^. However, the absorption of traditional metamaterial absorbers cannot be tuned once they are designed and fabricated. Recently, coherent perfect absorption (CPA)^19^ has been explored to achieve tunable absorption in a standing wave system formed by two counterpropagating beams. Such absorption can be coherently modulated in a range of 0–100% by changing the phase difference between two input beams^19^,^20^,^21^. It was recently reported that absorption^22^,^23^,^24^,^25^,^26^,^27^, polarization effects due to anisotropy and chirality^28^,^29^,^30^, all-optical computation^31^,^32^ in a thin metasurface can be controlled by a second wave incident on the same surface. The interaction of coherent light with the metasurface allows modulation of intensity^22^, polarization^29^ and propagation direction^30^ of light and also offers high contrast^32^, THz bandwidth^24^,^32^ and arbitrarily low intensity levels even down to single photon^33^. Coherent control promises new applications in space division multiplexing, image correction, 2D binary optical data processing and reconfigurable optical devices^32^.

Coherent light-matter interaction in ultrathin metamaterials has been demonstrated to dynamically modulate intensity, polarization and propagation direction of light^22^,^23^,^24^,^25^,^26^,^27^,^28^,^29^,^30^,^31^,^32^,^33^,^34^,^35^,^36^,^37^. Coherent control of metamaterials could be accomplished in various schemes, for instance, in the polarization standing wave^33^, from linear to circular polarization illumination^34^,^35^, from conventional metamaterials to gradient metasurfaces^30^ and from plasmonic to all-dielectric metasurfaces^36^. The gradient metasurface with a 2π transverse phase variation usually exhibits an anomalous refraction phenomenon. The incident beam will be divided into the normal and anomalous transmitted beams along two different directions in the far field. Generally, the input signal beam will be restricted to experience coherent modulation in two channels^30^. Less attention has been paid to the metasurface with multiple-order anomalous refracted beams^38^,^39^, which can provide a promising opportunity to realize multichannel signal modulation. It is worth investigating how this gradient metasurface is modulated in the coherent regime. Compared to the V-shaped and rectangular slit metasurfaces^30^, coherently controlled multiple anomalous modes offer a uniquely fertile ground for achieving all kinds of coherent optical devices, multi-functional plasmonic sensors and further understanding the interaction between gradient metasurfaces and the standing waves.

In this work, we investigate coherent control of multiple modes in a gradient metasurface constructed from an array of trapezoid apertures perforated through a freestanding gold film of substantially subwavelength thickness. The trapezoid-shaped slit metasurface produces multiple co-polarized anomalous reflected and transmitted beams. One normal and two deflected outgoing beams can be coherently controlled by changing the phase difference between two coherent beams. The gradient metasurface is an ultrathin freestanding gold nanostructure with continuously varying slit width in a super cell of trapezoid-shaped aperture, which can be practically fabricated from a gold-coated Si_3_N_4_ membrane using focused-ion-beam milling and reactive ion etching^32^.

## Results

### Metamaterial design and simulations

**Figure 1:**
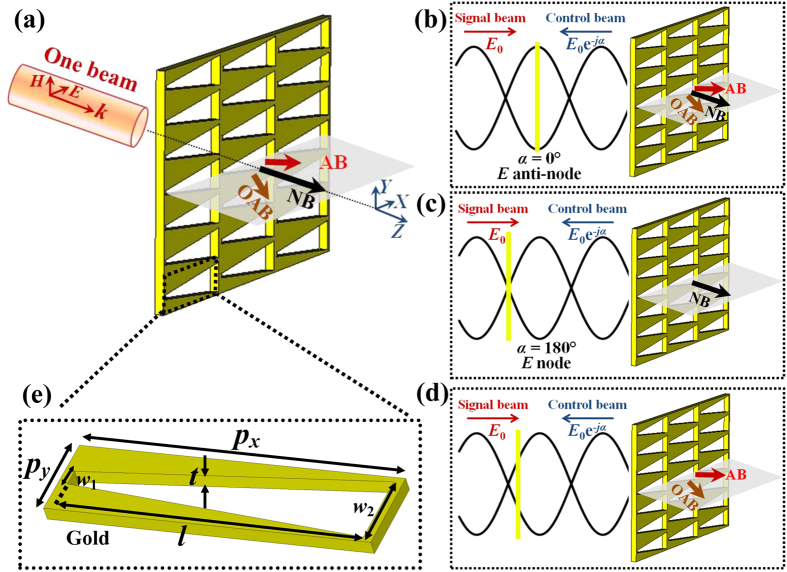
Schematic of the trapezoid-shaped slit metasurface. (**a**) Schematic of the anomalous and normal transmitted beams for a single *x*-polarized input beam incident on the metasurface along the *z* direction. (**b–d**) Schematic of coherent control of the trapezoid-shaped slit metasurface using coherent signal and control beams. Three different excitations occur when the metamaterial is located at an electric anti-node (**b**), an electric node (**c**) and a specific place of vanishing normal beam (**d**). (**e**) Structural details of the trapezoid-shaped slot antenna. The unit cell comprises a trapezoid-shaped slot antenna with a periodicity of *p*_*x*_ in the *x* direction and *p*_*y*_ in the *y* direction, generating a gradient phase shift along the *x* direction.

We propose a gradient metasurface with trapezoid-shaped slit building block that produces multimode diffraction while the trapezoid-shaped rod metamaterials were used to construct plasmonic absorber and spectrum splitter^38^,^40^. The negative metasurface is cut from an ultrathin gold film with a thickness of *t* = 50 nm. The unit cell comprises a trapezoid slot antenna with a periodicity of *p*_*x*_ in the *x* direction and *p*_*y*_ in the *y* direction (see Fig. 1), generating a gradient phase shift d*φ* along the *x* direction. A schematic of an optimized trapezoid slot antenna is given in Fig. 1(e), different from the multiple rectangular slit metasurface^30^. Here, *p*_*x*_ = 1200 nm and *p*_*y*_ = 300 nm. The trapezoid-shaped slit has a fixed length *l* = 1130 nm and variable widths. The minimal and maximal widths are *w*_1_ = 40 nm and *w*_2_ = 260 nm, respectively. This type of metasurface is known not to display polarization conversion^2^. For 0.5 < *λ*/*p*_*x*_ < 1 in the trapezoid-shaped slit metasurface, so there should be three diffraction orders exist^39^, the anomalous refraction, normal refraction and the opposite anomalous refraction as illustrated in Fig. 1(a). Such metasurface supports two deflected outgoing beams with opposite refraction angles. The two deflected beams possess the same linear polarization to both the incident wave and the normal outgoing beam and it is hard to tell one from the outgoing waves.

The proposed single trapezoid-shaped slot antenna continuously changes its width (40–260 nm) while its length (1130 nm) is larger than the operation wavelength. The trapezoid-shaped slit metasurface in the reflection mode will be a good candidate for introducing a 2π phase variation. Here, we mainly study the coherent absorption of the trapezoid-shaped slit metasurface in the transmission mode. In contrast to discrete phase variation from separate V-shaped or rectangular-shaped nanorods^1^,^2^, such a trapezoid-shaped slit in the super cell produces a quasi-continuous phase shift. According to the Babinet principle, the negative trapezoid-shaped slit has an additional phase shift *φ* along the metasurface’s interface for *x*-polarized incident light instead of *y*-polarized incident light. For an *x*-polarized single beam normally incident along the z direction, this gradient metasurface has co-polarized normal transmitted beams and anomalous refracted beams. The directions of anomalous *x*-polarized refracted beams are governed by the generalized Snell’s law as follows^1^,


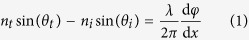


where *θ*_*i*_ and *θ*_*t*_ are the angles of incidence and transmission respectively and *λ* is the wavelength. Here we assume that transmission are into free space, so the refractive indices are *n*_*t*_ = *n*_*i*_ = 1.

**Figure 2:**
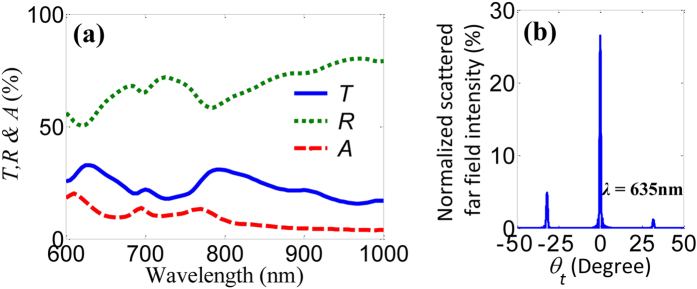
Simulated optical properties of the trapezoid-shaped slit metasurface for a single input beam. (**a**) Transmission *T*, reflection *R* and absorption *A* spectra. (**b**) Normalized far field intensity as a function of refraction angle *θ*_*t*_ at the wavelength *λ* = 635 nm.

Based on full-wave simulations using a full three-dimensional Maxwell finite element method solver^41^, the total transmitted, reflected and absorbed intensities are shown in Fig. 2(a), where the permittivity of gold was calculated by the Drude-Lorentz model^42^. The trapezoid-shaped slit metasurface exhibits broadband anomalous refraction as well as anomalous reflection. The total transmission (reflection) includes the output intensities of all the anomalous and normal transmitted (reflected) beams. At a wavelength of *λ* = 635 nm, the total transmitted and reflected intensities (*T* and *R*) are about 32.3% and 55.4% while the absorption *A* is about 13.3%. However, we cannot easily characterize the intensities and directions of the anomalous beams from this metasurface since its polarization is identical to that of the normal beam. By taking the Fourier transform of the output electric field, we calculate the far field pattern to show the directions and intensities of the normal and anomalous beams. The far field intensities are normalized to the free space without the metasurface. It is clearly found that the total output intensities include three different beams, one normal beam and two deflected beams as illustrated in Fig. 2(b). The normal beam propagates along the z axis with intensity of ~26.4% while the anomalous beam can be redirected to the right side with deflection angle of ~−32° and intensity of 5%. Particularly, the other weak diffraction mode cannot be ignored and redirected to the left side with deflection angle of ~32° and intensity of 1.1%, so-called opposite anomalous beam^39^. Compared to previous metasurfaces^1^,^2^,^3^,^4^,^5^,^6^,^7^,^8^,^9^,^10^,^39^, the opposite anomalous beam can be greatly enhanced by trapezoid-shaped slit structure. The calculated values of the total far field intensity are in a good agreement with the FEM simulation shown in Fig. 2(a).

### Coherent control of two deflected anomalous beams

The normal and anomalous beams produced by gradient metasurfaces can be coherently modulated and switched on/off by simply changing the phase difference. The trapezoid-shaped slit metasurface discussed in this work exhibits multiple modes. It is interesting to know how the responses of these modes evolve in the standing wave interference pattern formed by two counterpropagating beams. The output property from the metasurface is actually determined by its position in this standing wave, or equally a relative phase difference *α* between two coherent beams. In the limiting cases, a gradient metasurface of substantially subwavelength thickness can be placed either at an electric anti-node or node, leading to enhanced or vanishing electric excitation, and therefore scattering, respectively. The coherently controlled schematic of the trapezoid-shaped slit metasurface is illustrated in Fig. 1(b), (c) and (d). At specific place, the normal beam can be completely suppressed due to destructive interference while two anomalous beams deflect along different angles shown in Fig. 1(e). The output of the signal beam can be modulated by another coherent control beam.

**Figure 3:**
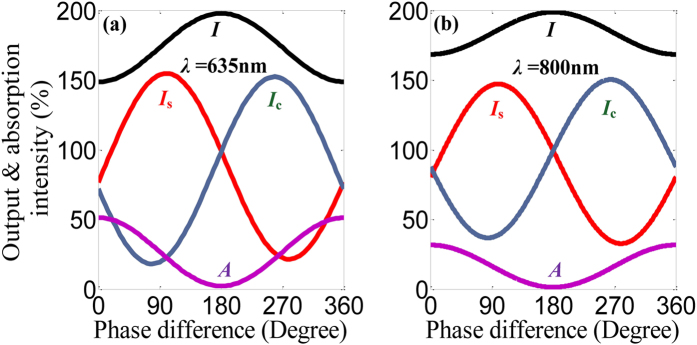
Coherent control of the trapezoid-shaped slit metasurface. Total output *I*, signal output *I*_s_ and control output *I*_c_ intensities and coherent absorption *A* of the gradient metasurface are shown at the wavelengths *λ* = 635 nm (**a**) and *λ* = 800 nm (**b**) as a function of the phase difference *α* between the control and signal beams.

The input intensities of the signal and control beams are defined as 100% each and thus the total output intensity is 200% without any absorption. *I*_s_ and *I*_c_ denote output intensities of signal and control beams, respectively. The total output intensity *I* is a sum of *I*_s_ and *I*_c_ (*I* = *I*_s_ + *I*_c_), while the absorbed energy is *A* = 2 − *I*. The scattering and absorption properties of the trapezoid-shaped slit metasurface at two selective wavelengths of *λ* = 635 and 800 nm are shown in Fig. 3. The coherently controlled metasurface behaves somewhat a beam splitter and reveals sinusoidal dependent output intensities. The energy can transfer between *I*_s_ and *I*_c_. Apparently, the intensities of the output beams and the total absorption depend strongly on the phase difference between the control and signal beams. As the phase difference *α* varies in a range of 0° to 360°, the coherent absorption in the metasurface is modulated between 51.2% and 2.2% while the corresponding total output intensity *I* changes between 148.8% and 197.8% at *λ* = 635 nm. Advantageously, the trapezoid-shaped slit metasurface exhibits much lower absorption due to large air apertures. Importantly, this coherently controlled mechanism can be applied to metasurfaces in a broadband wavelength range. Another wavelength is picked up to verify such feature. At *λ* = 800 nm, the coherent absorption can be coherently controlled between 31.6% and 1.4% and the total output intensity *I* changes between 168.4% and 198.6%. The coherently controlled metasurface has fourfold enhanced absorption compared to single beam illumination when excited in-phase at an electric anti-node of the standing wave (i.e., *α* = 0°), while zero absorption when excited out-of-phase at an electric node (i.e., *α* = 180°). The absorption of materials can be completely suppressed using the coherent technique, which is significant for developing high-efficient coherent photonic devices.

**Figure 4:**
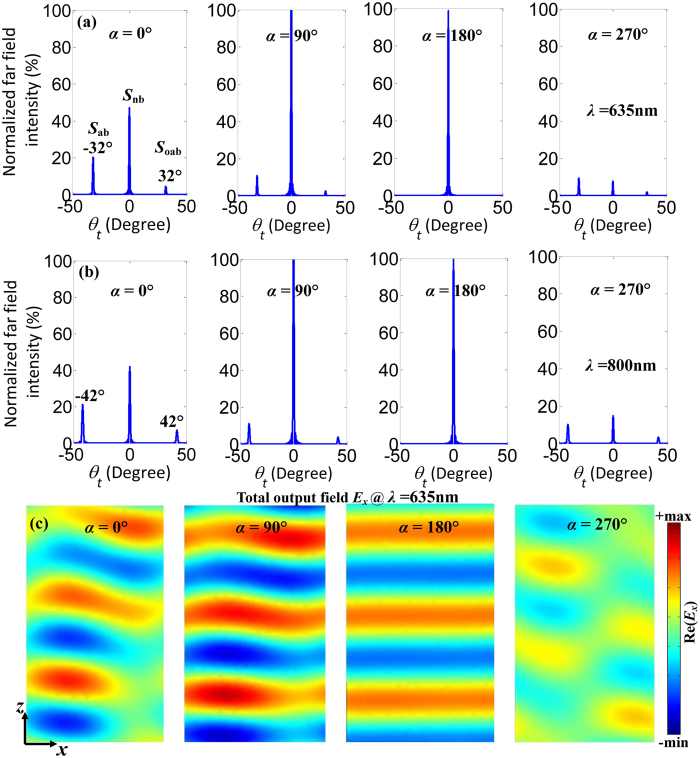
Coherent control of the gradient metasurface with trapezoid-shaped slot antenna. Normalized far field intensity as a function of refraction angle *θ*_*t*_ for the phase difference *α* = 0°, 90°, 180° and 270° at *λ* = 635 nm (**a**) and *λ* = 800 nm (**b**). (**c**) *E*_*x*_ field patterns of the signal output for *α* = 0°, 90°, 180° and 270°. All color maps are on the same scale.

The scattering intensities from the normal, anomalous and opposite anomalous beams contribute to both *I*_s_ and *I*_c_ in Fig. 1(b). Identically, *I*_s_ = *I*_nb_ + *I*_ab_ + *I*_oab_, where *I*_nb_, *I*_ab_ and *I*_oab_ are scattering intensities from the normal, anomalous and opposite anomalous beams. The scattering directions of three separated beams are kept constant in the coherent case, but the scattering intensities *I*_nb_, *I*_ab_ and *I*_oab_ strongly depend on the phase difference in Fig. 4. At an electric anti-node of the standing wave, the trapezoid-shaped slit metasurface is excited in-phase (i.e., *α* = 0°) and accordingly scattering intensities of the anomalous and opposite anomalous output beams increase. In comparison to output intensities for single beam excitation, the output intensities of anomalous and opposite anomalous beams increases fourfold to *I*_ab_ = 20.2% and *I*_oab_ = 4.5% at *λ* = 635 nm while *I*_ab_ = 21.2% and *I*_oab_ = 7.1% at *λ* = 800 nm in the coherent control case, respectively. For anti-phase excitation at an electric node of the standing wave (i.e., *α* = 180°), the interaction of the metasurface with the standing wave is negligible, thus the output intensities *I*_ab_ and *I*_oab_ of the anomalous and opposite anomalous beams are completely suppressed to zero and the normal output beam intensity *I*_nb_ is nearly 100%. In contrast to the intensity, the propagation directions of the two deflected beams are insensitive to the phase difference *α* between the control and signal beams. There are always 32° and 42° deflection away from the normal for anomalous and opposite anomalous beams at *λ* = 635 and 800 nm in Fig. 4(a) and (b), respectively. This is because the coherent control process controls the overall level of scattering from the structure but does not change the phase gradient imposed by the metasurface, which is determined solely by the metasurface design. This allows simple modulation of the output beam intensities through phase control without distorting or redirecting the individual beams. Figure 4(c) presents the total signal output field patterns *E*_*x*_ of the gradient metasurface for *α* = 0°, 90°, 180°and 270° at *λ* = 635 nm. It is clearly found that the wave front of the output beam changes with the phase difference *α* and the output is not a plane wave except for *α* = 180°. When *α* = 180°, the signal output beam is a perfect plane wave due to vanishing anomalous beams, consistent with Fig. 4(a). Therefore, the wave front of light can be coherently modulated in the gradient metasurface. The signal beam has three channel outputs that can be coherently modulated in terms of coherent absorption. Interestingly, this coherent control technique can be applied over a broad range of wavelengths and promises many interesting applications such as wave front shaping and multichannel signal routing.

**Figure 5:**
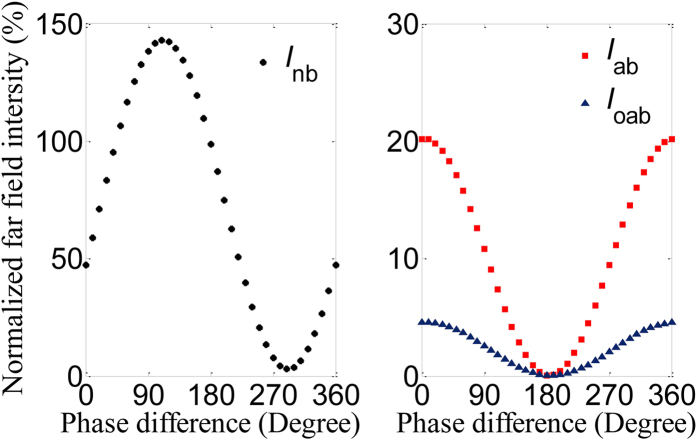
Coherent control of multiple modes in the gradient metasurface with trapezoid-shaped slot antenna. Normalized far field intensity of the normal beam (**a**) and two anomalous beams (**b**) as a function of the phase difference *α* for *λ* = 635 nm.

In fact, according to the simulated results in Figs 3 and 4 we cannot thoroughly understand how three output modes behave as the phase difference varies. Especially, anomalous and opposite anomalous modes are unknown in the coherent case. In order to visualize the coherent control of multiple modes, their normalized scattering far field intensities are presented as a function of the phase difference in Fig. 5. Here, the calculated results of the far field intensities are consistent with the FEM simulations shown in Fig. 3. In Fig. 5(a), the normalized scattering far field intensity *I*_nb_ of the normal beam varies in the range of 3%–143% when the phase difference changes over the 2π phase range. The maximal and minimal values of *I*_nb_ individually emerge for *α* = 110° and 290°. Obviously, the scattering output intensities *I*_ab_ and *I*_oab_ of the anomalous and opposite anomalous beams are synchronously dictated by the phase difference as shown in Fig. 5(b). Their maximal and minimal output intensities always correspond to *α* = 0° and 180°, respectively. *I*_ab_ of the anomalous beam decrease from 20.2% to 0 while correspondingly *I*_oab_ of the opposite anomalous beam decrease from 4.5% to 0 when the phase difference changes from 0° to 180°, which is different from the coherently controlled normal beam. Therefore, we can distinguish the anomalous and opposite anomalous beams from the normal beam based on the coherent technique. Remarkably, we can recognize the coherently controlled behaviors of the two anomalous modes.

**Figure 6:**
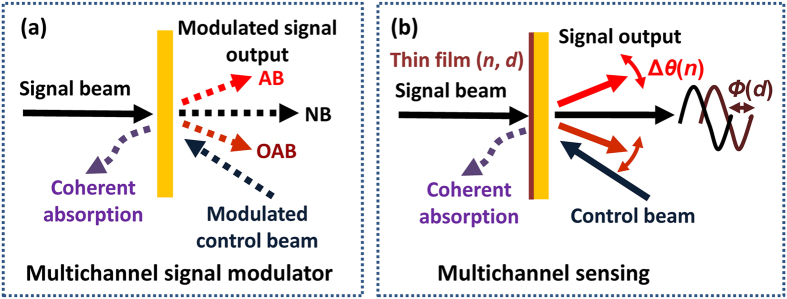
Illustrations of potential applications. (**a**) Coherent multichannel signal modulator. An intensity- or phase-modulated control beam will govern signal output. (**b**) Coherent multichannel sensors. The deflected angle change Δ*θ* of two anomalous beams is directly linked to the refractive index of the sensing film and the output of the normal beam exhibits the phase shift *ϕ* in the coherent case that depends on its thickness.

The interaction between ultrathin metamaterials and the standing wave provides various potential applications from coherent optical devices, coherent signal processing to coherent spectroscopy^22^. Besides perfect transparency and total absorption, the coherent technique leads to near-perfect switching effect of anomalous beams in the gradient metasurface based on coherent absorption and interference effect. The trapezoid-shaped slit metasurface with multiple modes is promising for multichannel signal processing and sensing and the corresponding schemes are shown in Fig. 6. Coherent absorption can enable one to realize analog and digital, all-optical modulation/switching^22^. When the phase or intensity-modulated control beam is applied, all outgoing AB, NB and OAB from the trapezoid-shaped slit metasurface can be coherently modulated. Consequently, multichannel signal modulator can be realized in Fig. 6(a). The slit metasurface is readily filled with sensing medium, therefore facilitating the detection of ambient changes. Another potential application of the trapezoid-shaped slit metasurface is multichannel sensing in Fig. 6(b). It is sensitive to ambient thin film or liquid with its refractive index *n* and thickness *d*. Obviously, the deflected angle change Δ*θ* of two anomalous beams is directly linked to the refractive index of the thin film according to the generalized Snell’s law, while the output of the normal beam exhibits the phase shift *ϕ* in the coherent case that depends on the film optical thickness. Thus the sensing medium can be coherently identified by detecting far field signal outputs from the trapezoid-shaped slit metamaterial, provided that the location of metasurface is accurately adjusted. The proposed metasurface may serve practical applications in multichannel sensors and signal modulation.

## Discussion

In summary, we have proposed a single trapezoid-shaped slit metasurface with three discrete transmitted beams that are normal beam, anomalous and opposite anomalous beams. It is demonstrated for the first time that output properties of multiple modes in the ultrathin metasurface can be coherently controlled by use of the relative phase difference between two coherent input beams. One normal and two deflected beams have sinusoidal dependent output intensities and the energy can transfer between the signal and control output. Particularly, each mode can be completely switched on/off and two deflected beams are synchronously dictated by changing the phase difference. In the coherent control regime, although the propagation directions of three output beams remain unaltered, the wave front may be coherently controlled based on metasurfaces. Coherently controlled metasurfaces promise new applications including multichannel signal routing, wavefront shaping, data processing, mode selection and surface wave manipulation^43^.

## Methods

Based on full-wave simulations using a full three-dimensional Maxwell finite element method solver^41^, the total transmitted, reflected and absorbed intensities of the metasurface were calculated, where the permittivity of gold was calculated by the Drude-Lorentz model^42^. By taking the Fourier transform of the output electric field, the far field patterns were calculated to show the directions and intensities of the normal and anomalous beams. The far field intensities were normalized to the free space without the metasurface. The input intensities of the signal and control beams are defined as 100% each and thus the total output intensity is 200% without any absorption.

## Additional Information

**How to cite this article**: Zhu, Z. *et al.* Coherent control of double deflected anomalous modes in ultrathin trapezoid-shaped slit metasurface. *Sci. Rep.* **6**, 37476; doi: 10.1038/srep37476 (2016).

**Publisher's note:** Springer Nature remains neutral with regard to jurisdictional claims in published maps and institutional affiliations.
